# Changes in working memory performance and cortical activity during acute aerobic exercise in young adults

**DOI:** 10.3389/fnbeh.2022.884490

**Published:** 2022-08-02

**Authors:** Kefeng Zheng, Zhangyan Deng, Jiali Qian, Yanxia Chen, Shiyuan Li, Tao Huang

**Affiliations:** ^1^Department of Physical Education, Shanghai Jiao Tong University, Shanghai, China; ^2^School of Education, Shanghai Jiao Tong University, Shanghai, China

**Keywords:** exercise, working memory, cortical activity, fNIRS, young adults

## Abstract

This study aimed to examine the concurrent performance of working memory and cortical activity during acute aerobic exercise in young adults. In a crossover study design, 27 young adults (mean age = 22.7 ± 3.4 years, 15 women) participated in two experimental conditions in a randomized order: (1) sitting condition (without exercise) and (2) cycling condition (moderate-intensity exercise). Working memory was measured with a modified version of the n-back task. A functional near-infrared spectroscopy (fNIRS) was used to measure cortex activation. In the cycling condition, response time (RT) for the n-back task was significantly faster (*p* < 0.05). No differences in accuracy were observed between the sitting and cycling conditions. The fNIRS results showed that the oxygenated hemoglobin (oxy-Hb) concentrations in the bilateral frontopolar area (*p* < 0.05), dorsolateral prefrontal cortex (*p* < 0.05), and right premotor and supplementary cortex (*p* < 0.05) were decreased while cycling. The findings indicated that the concurrent performance of working memory was improved during acute aerobic exercise, whereas cortical activity was decreased in some brain regions.

## Introduction

Working memory is not only crucial to scholastic performance in childhood (Hitchcock and Westwell, 2017; Spiegel et al., 2021), but it is also linked with mental health in adulthood (Nikolas et al., 2019; Morales-Munoz et al., 2021) and successful aging (Bosnes et al., 2022). The prefrontal cortex (PFC) plays an important role in modulating higher-level functions such as working memory (Robbins and Arnsten, 2009; McGuire and Botvinick, 2010). Previous studies have shown that chronic exercise may improve working memory over the lifespan (Padilla et al., 2014; Xue et al., 2019; Liu et al., 2020; Ludyga et al., 2022). Few studies have assessed working memory performance during acute exercise. Clarifying how working memory performance is influenced during physical exertion has practical implications. For instance, successful sports performance, especially in open-skill exercise, highly depends on the ability to exercise concurrently while undergoing cognitive demands (Davranche et al., 2005). Similarly, some high-stress occupations (e.g., firefighter or military personnel) involve higher-order cognitive processes (e.g., working memory) while simultaneously engaging in physical efforts (Stone et al., 2020).

Arousal level may be associated with cognitive performance (Lambourne and Tomporowski, 2010; Munn et al., 2021). An optimal level of arousal may be induced by moderate-intensity exercise, thereby enhancing cognitive performance (Brisswalter et al., 2002; Tomporowski, 2003). Meanwhile, according to catecholamine hypothesis of exercise-cognition interaction, moderate-intensity exercise may facilitate cognitive performance (McMorris, 2021). However, existing studies yielded mixed findings regarding working memory performance during exercise. For instance, Martins et al. (2013) and Komiyama et al. (2017) found that working memory performance was improved during moderate-intensity exercise in young adults. In contrast, Audiffren et al. (2009) and Lambourne et al. (2010) suggested that working memory performance was maintained at the same level.

Most of the aforementioned studies were limited to behavioral measurements, whereas the cognitive task-evoked cortical activity associated with working memory during exercise was unclear. Previous neuroimaging studies, primarily using functional magnetic resonance imaging (fMRI) (Ding et al., 2021; Domingos et al., 2021) and electroencephalogram (EEG) (Chang et al., 2017; Silveira et al., 2019; Wu et al., 2019), have examined cognitive task-evoked neural activation after exercise intervention. However, the in-task cortical activity during exercise was less investigated. The neuroimaging technique functional near-infrared spectroscopy (fNIRS) provides non-invasive, portable measurements of cortical activation. It is relatively robust with motion artifacts, so it can be used to measure cortical hemodynamics under physically demanding conditions (Tempest and Reiss, 2019). Some available fNIRS studies suggested that cortical activation pattern changed when conducting cognitive tasks during exercise. One of our previous studies (Huang et al., 2019) examined the effects of self-paced low-intensity cycling on inhibitory control, cognitive flexibility, and cortical activation in young adults. The fNIRS results showed that the cycling condition resulted in lower oxygenated hemoglobin (oxy-Hb) concentrations associated with the Stroop interference effects in some PFC regions. In contrast, oxy-Hb concentrations associated with global switch costs were higher during cycling. An fNIRS study by Tempest and Reiss (2019) found that increased activation of the PFC associated with a working memory task during cycling. Therefore, further studies are needed to investigate the cognitive task-evoked cortical activity during exercise.

Taken together, changes in working memory performance and underlying cortical activation patterns during acute aerobic exercise remain unclear. Therefore, the purpose of this study was to investigate the performance of working memory and task-evoked cortical activity during acute aerobic exercise.

## Methods

### Participants

Twenty-nine right-handed young adults voluntarily took part in this study. All the participants were recruited from Shanghai Jiao Tong University. No participants reported any cardiovascular diseases, mental illnesses, or neurological disorders. They were provided with informed consent forms before the experiment and full information on the protocol of the study. The protocol was reviewed and approved by the ethical committee of Shanghai Jiao Tong University (ethical code: H2020042I).

Sample size was determined using the G^*^Power software (Faul et al., 2007). Statistical power and sample size calculations (α = 0.05, 1-β = 0.80) were performed based on the effect size (Cohen's d = 0.55) of the study by Tempest and Reiss (2019). A total of 18 participants were required to achieve a power of 80% to detect a significant within-group difference. Finally, 29 participants volunteered to participate in this study. Two participants were excluded from data analyses because of non-compliance with the behavioral measurement, yielding a final sample of 27 participants (mean age 22.7 ± 3.4 years, 15 women). Additionally, six participants were discarded from the fNIRS analysis because of insufficient signal quality, resulting in 21 participants for the fNIRS analysis.

### Experimental protocols

This study used a randomized crossover design. The participants visited the laboratory on three separate occasions. On the first visit, the participants' handedness was verified using a validated Chinese version of the Handedness questionnaire (Li, 1983). The participants completed a physical activity readiness questionnaire (PAR-Q) to ensure safety for the following assessment. In an incremental cycle ergometer test (Ergomedic 839E; Monark Exercise AB, Varberg, Sweden), maximal aerobic power (MAP) was measured in order to determine the individual exercise workload of the following moderate-intensity exercise protocol (50% MAP). An initial workload of 25 W was set, and the workload was gradually increased by 25 W every 2 min. The participants were advised to remain at or above 60 revolutions per minute (RPM) throughout the test. After exhaustion or when a participant could not maintain 60 RPM, the test was terminated. Heart rate (HR) was determined using a Suunto heart rate monitor (SuuntoM5; SUUNTO Inc., Vantaa, Finland). The Borg Rating of Perceived Exertion (RPE) Scale was used to measure self-perceived physical exertion (Borg, 1998). HR and RPE were recorded following each increment. The participants were verbally encouraged to achieve their maximum level, particularly once they reported an RPE of 17 or higher.


(1) $$\begin{array}{l} {\text{W}}_{\text{max}}\;=\;{\text{W}}_{\text{com}}+\frac{\text{t}}{120}*25, \end{array}$$

**Figure 1:**
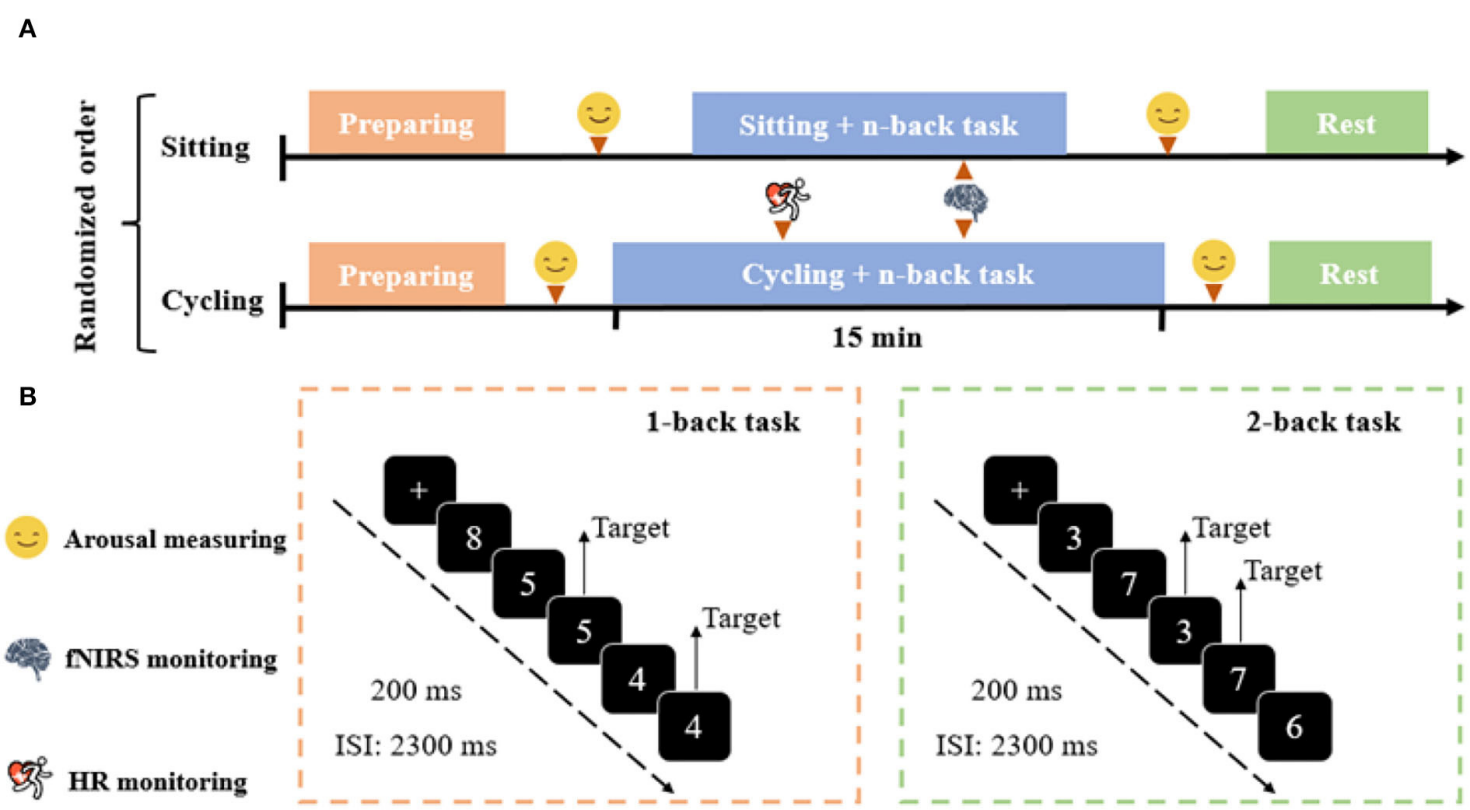
Experimental protocol. **(A)** Experimental design consisting of the sitting and cycling conditions. **(B)** Schematic illustration of the n-back task. Examples of trials for the 1-back and 2-back conditions of the n-back task are exemplified.

MAP (W_max_) was calculated with the equation above (Kuipers et al., 1985), where W_com_ represents the load of the last completed stage, and t (second) represents the time of the last incomplete stage. There was a 48-h interval between the MAP test and the first experimental session. The next two visits involved the sitting condition (without exercise) and the cycling condition (moderate-intensity exercise), which were conducted with a counterbalanced measure design (Figure 1A). Each condition occurred 7 days apart but at the same time of the day. For the cycling condition, the total duration consisted of 15 min of exercise on a cycle ergometer. The exercise began with a 3-min warmup in which the participants reached the desired steady-state heart rate (~64–76% HR_max_). Then, the participants were required to maintain this intensity throughout the n-back task. The n-back task was performed at the fourth minute following the onset of exercise and lasted ~8 min. There was a 15-s “cognitive rest” interval between each block during which the participants continued to cycle. Finally, there was a 3-min active rest at a 30-W workload to bring the total cycling time to 15 min for each participant. The participants' HR and cortical activation were continuously monitored throughout the test. RPE and arousal levels were recorded immediately after exercise. The sitting condition used a similar procedure. The participants were required to perform the n-back task when sitting on the cycle ergometer without cycling. It is noteworthy that the sitting condition was shorter than the cycling condition because there was no warmup and an active-rest period.

### Psychological measures

The Felt Arousal Scale (FAS) was adopted to measure arousal level changes (Svebak and Murgatroyd, 1985). The scale ranges from 1 (low arousal) to 6 (high arousal). In this study, the participants were asked to rate their present psychological state before and immediately after the experimental conditions (pre and post-sessions).

### n-back paradigm

This study employed modified versions of the n-back task (Kao et al., 2020), a well-known paradigm for investigating working memory. The task was programmed and presented with the E-Prime 3.0 software (Psychology Software Tools Inc., United States). The number of stimuli (between 3 and 8) was displayed in the center of the screen (Figure 1B). The n-back task consisted of eight blocks of 96 trials. Each block included 12 trials (four target and eight non-target trials) presented in a pseudorandomized order. In each trial, a single number was presented in the center of a computer screen for 200 ms, followed by an inter-stimulus interval (ISI) of 2,300 ms. The participants should respond within 1,000 ms. Responding with a left-hand press (“F” key) was required when the current stimulus matched the stimulus from n-steps earlier in the sequence (i.e., target), and with the right-hand press (“J” key) when the current stimulus did not match the n steps earlier in the sequence (i.e., non-target). For instance, in the 2-back task condition, the participants were required to use their left hand to respond to the same number presented two trials earlier, and with their right hand to respond to all other numbers. Each task block began with a 5-s cue that informed of the following task condition, and the task blocks were separated with 15-s resting periods. Each task block lasted 35 s, and the whole session lasted ~8 min. The participants were required to respond as quickly and accurately as possible. Response time (RT) and accuracy were recorded. An initial practice block was administered with 1-back and 2-back conditions before the experiment commenced to ensure that the participants understand the task.

### fNIRS measurement

**Figure 2:**
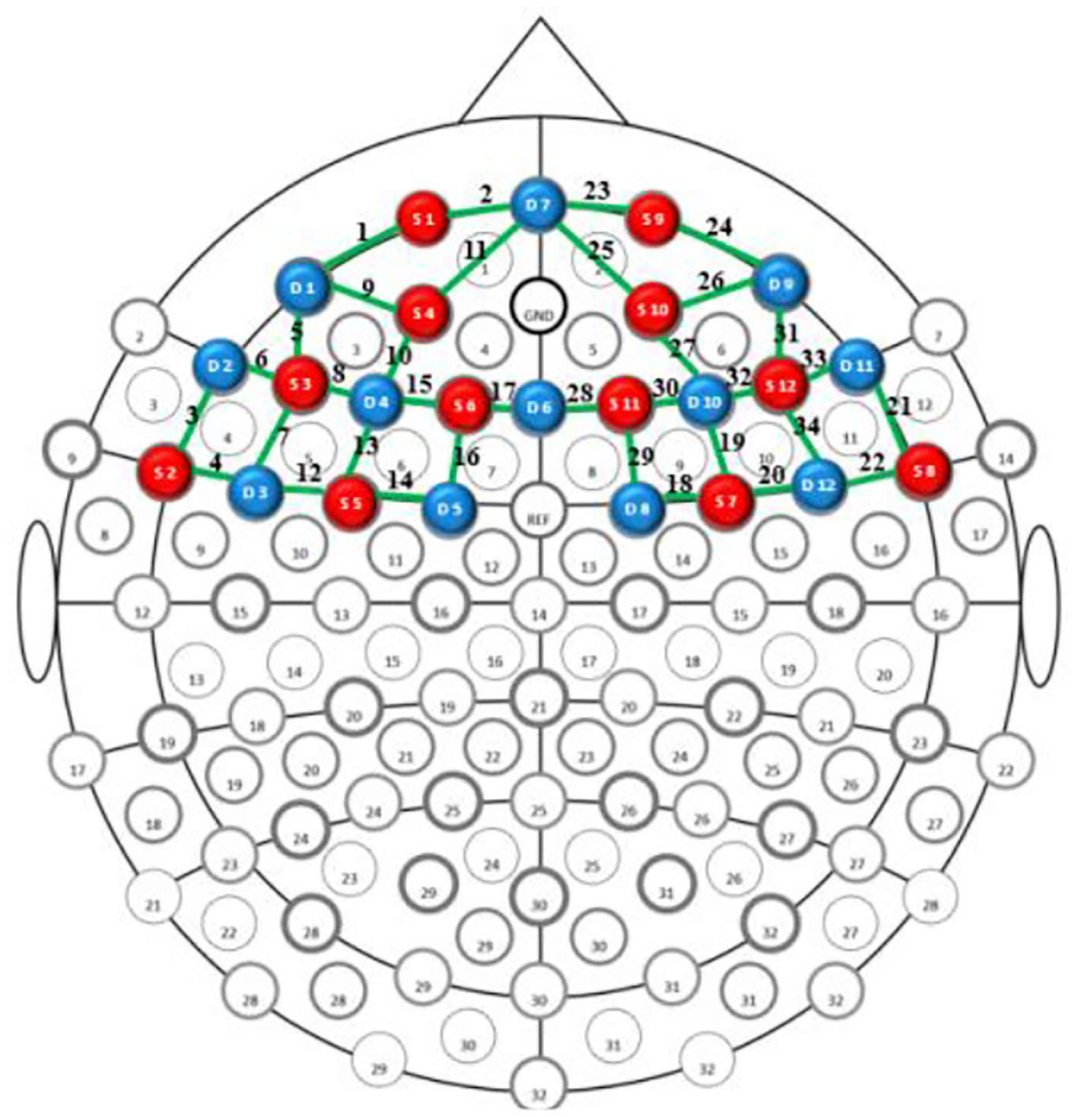
fNIRS probe layout. The red and blue circles indicate sources and detector probes, respectively. Green lines represent the nearest source-detector pairs (channels).

During the two experimental conditions, cortical hemodynamic changes were monitored using a multi-channel continuous-wave fNIRS system (NIRSport 2; NIRx Medical Technologies, United States) at a sampling rate of 6.78 Hz. The montage setup consisted of 12 dual-wavelength (760 and 850 nm) source probes, and 12 optical detector probes covered several 10–10 electroencephalography positions. By arranging the 12 sources and the 12 detector probes alternately at a distance of 3 cm, there are 34 channels (Figure 2). The probability of estimating spatial information (Supplementary Table 1) for each channel of a brain region was determined using the probabilistic estimation method (Okamoto et al., 2004; Tsuzuki et al., 2007). Optical density data were analyzed using the modified Beer-Lambert Law to calculate signals reflecting oxy-Hb, deoxygenated hemoglobin (deoxy-Hb), and total hemoglobin (total-Hb) signal changes. Compared to deoxy-Hb and total-Hb signals, oxy-Hb signals have a higher signal-to-noise ratio (Strangman et al., 2002) and retest reliability (Plichta et al., 2006). Therefore, this study used oxy-Hb signals as indicators of regional cortex activity.

### fNIRS data analysis

The raw data of fNIRS were preprocessed using NIRSLAB (NIRx, United States). A bandpass filter (in the 0.01 to 0.5 Hz range to remove noise like respiratory frequency, low-frequency oscillations, and Mayer waves) was applied, and the data were converted to oxy-Hb concentrations. The mean values of the oxy-Hb concentrations from the baseline (0–2 s before the onset of the trial) and the task (4–50 s after the onset of the trial) were computed for each participant, region of interest (ROI), and task condition.

**Figure 3:**
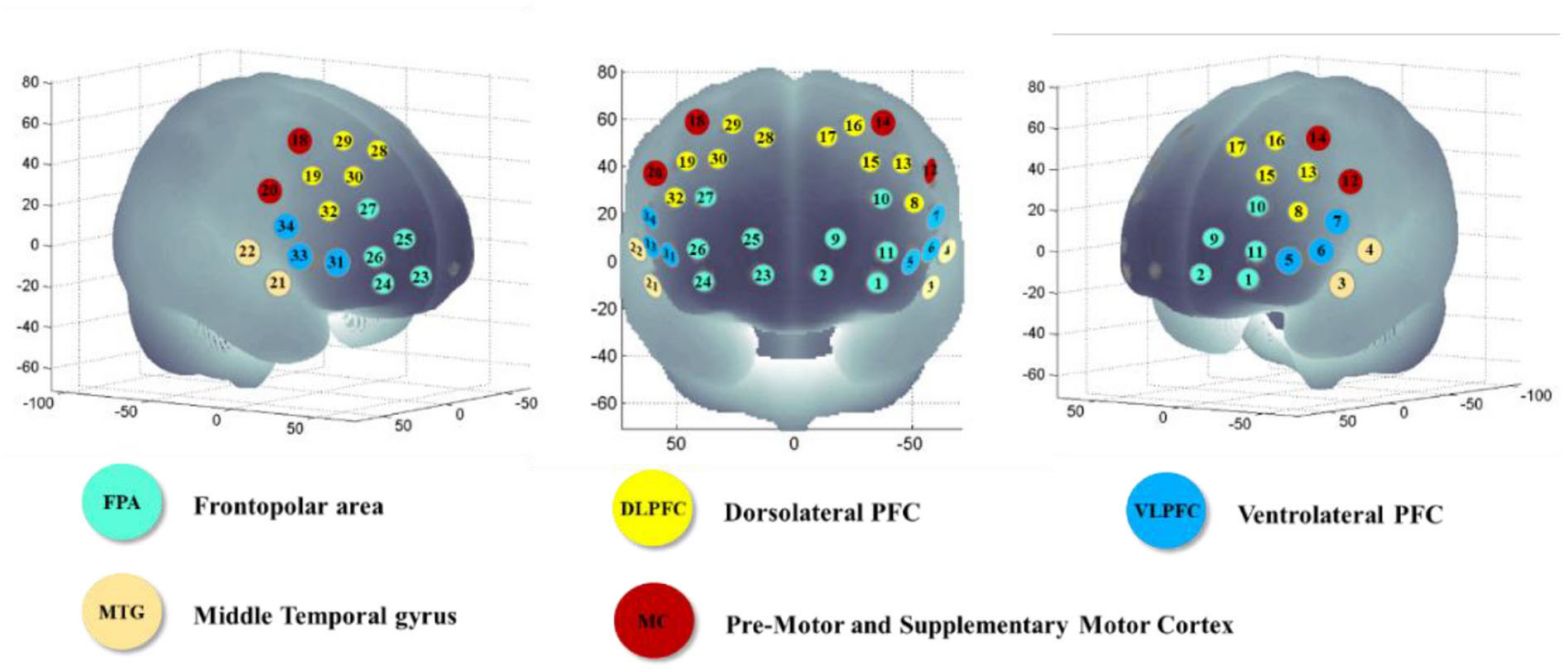
Localization of each channel, Montreal Neurological Institute (MNI) coordinates, and Brodmann areas.

In previous studies, some brain regions were activated during similar motor (bilateral motor cortex) and n-back (bilateral PFC) tasks (Tempest and Reiss, 2019). Thus, the ROIs in this study included the bilateral frontopolar area (FPA), dorsolateral prefrontal cortex (DLPFC), ventrolateral prefrontal cortex (VLPFC), middle temporal gyrus (MTG), and pre-motor and supplementary cortex (MC) regions. For ROI definition, this study used the automated anatomical labeling atlas Brodmann (Rorden and Brett, 2000). Regions included the left (channels 1, 2, and 9–11) and right (channels 23–27) FPAs, left (channels 8, 13, and 15–17) and right (channels 19, 28–30, and 32) DLPFCs, left (channels 5–7) and right (channels 31, 33, and 34) VLPFCs, left (channels 3 and 4) and right (channels 21 and 22) MTGs, and left (channels 12 and 14) and right (channels 18 and 20) MCs (Figure 3).

### Statistical analysis

Statistical analyses were performed using SPSS V.22 (IBM, Chicago, IL, United States). The RT and accuracy of the n-back task and oxy-Hb signal changes in all the ROIs were analyzed by 2 (experimental condition: sitting and cycling) × 2 (task condition: 1-back and 2-back) repeated-measures analysis of variance (RM-ANOVA). Spearman's correlation coefficients were calculated to evaluate the relationship between exercise-induced arousal and n-back task performance. Partial eta squared ($\eta_{\text{p}}^{2}$) was calculated as a measure of effect size. Statistical significance levels were set at *p* < 0.05. False discovery rate (FDR) was used to control for multiple testing for ROI-wise analyses of the fNIRS data.

## Results

### Physiological and psychological parameters

**Figure 4:**
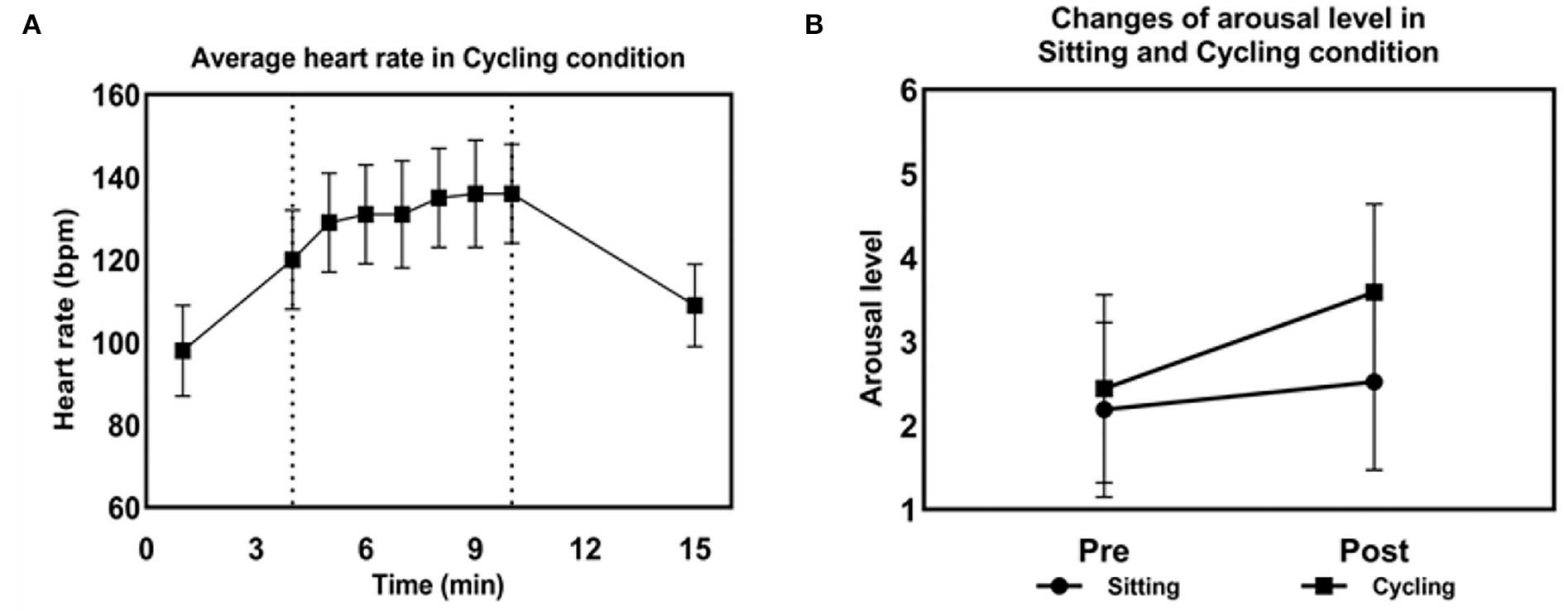
Physiological and psychological parameters during the cycling condition. **(A)** Changes in heart rate in the cycling condition. **(B)** Changes in arousal levels in the sitting and cycling condition.

The changes in heart rate and arousal level are presented in Figure 4. The mean of HR and RPE in the cycling condition was 131.14 ± 11.55 beats/min (~73% HR_max_) and 13.59 ± 1.15 points, respectively. The two physiological parameters showed that the exercise intensity of cycling condition was moderate based on the American College of Sports Medicine (ACSM) guidelines (Garber et al., 2011).

### Behavioral results

**Figure 5:**
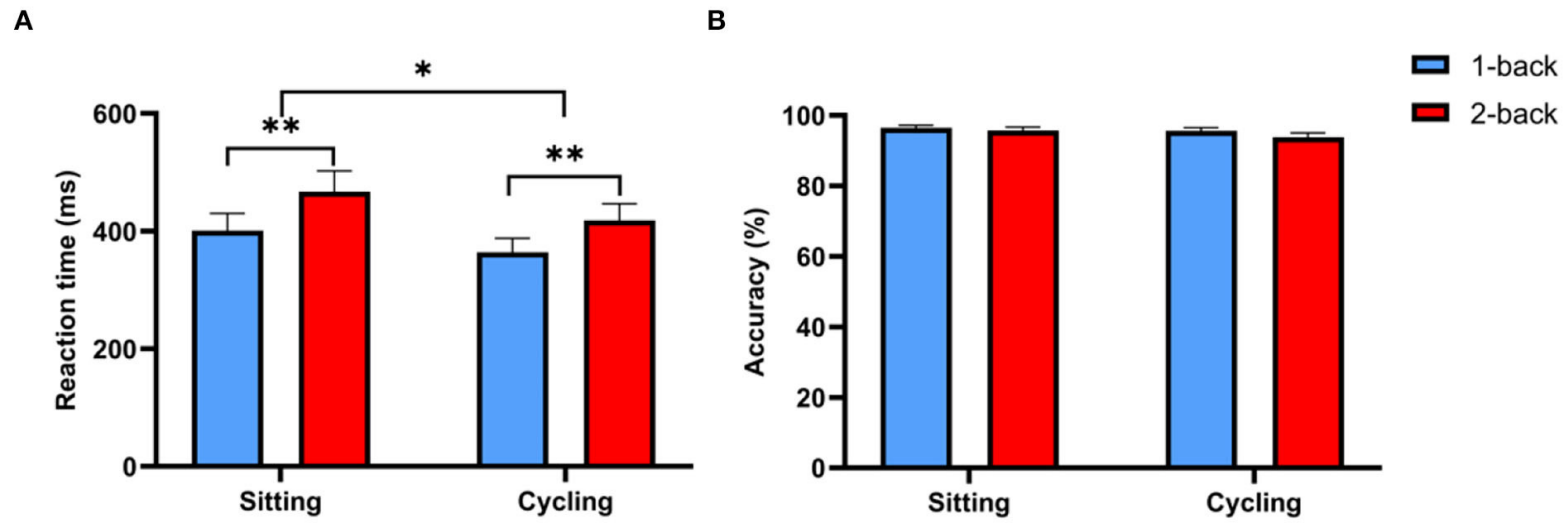
RT and accuracy of the n-back task. **(A)** Comparison of RT between the sitting and cycling conditions. **(B)** Comparison of accuracy between the sitting and cycling conditions. Data are expressed as mean ± standard error. ^*^ indicates *p* < 0.05; ^**^ indicates *p* < 0.001.

For RT, as shown in Figure 5, the RM-ANOVA revealed that the main effects of the task and experimental conditions were both significant [*F*_(1,26)_ = 30.72, $\eta_{\text{p}}^{2}$ = 0.54, *p* < 0.001, and *F*_(1,26)_ = 4.78, $\eta_{\text{p}}^{2}$ = 0.16, *p* < 0.05, respectively]. The RT of the 1-back task was significantly faster than that of the 2-back task. In the sitting condition, the RT of the 1-back task was significantly faster than that of the 2-back task (400.96 ± 29.17 ms vs. 467.3 ± 35.11 ms, *p* < 0.001). Accuracy was not significantly different between the 1-back and 2-back tasks (96.46 ± 0.74% vs. 95.74 ± 0.95%, *p* > 0.05). In the cycling condition, the RT of the 1-back task was significantly faster than that of the 2-back task (363.85 ± 24.12 ms vs. 418.48 ± 28.24 ms, *p* < 0.001). There were no significant differences in accuracy between the 1-back and 2-back tasks (95.62 ± 0.93% vs. 93.8 ± 1.23%, *p* > 0.05). The RT of the cycling condition was significantly faster than that of the sitting condition (391.17 ± 25.26 ms vs. 434.13 ± 31.56 ms, *p* < 0.05). The RM-ANOVA performed on the accuracy of the n-back task revealed that there were no significant main or interaction effects.

### fNIRS results

**Figure 6:**
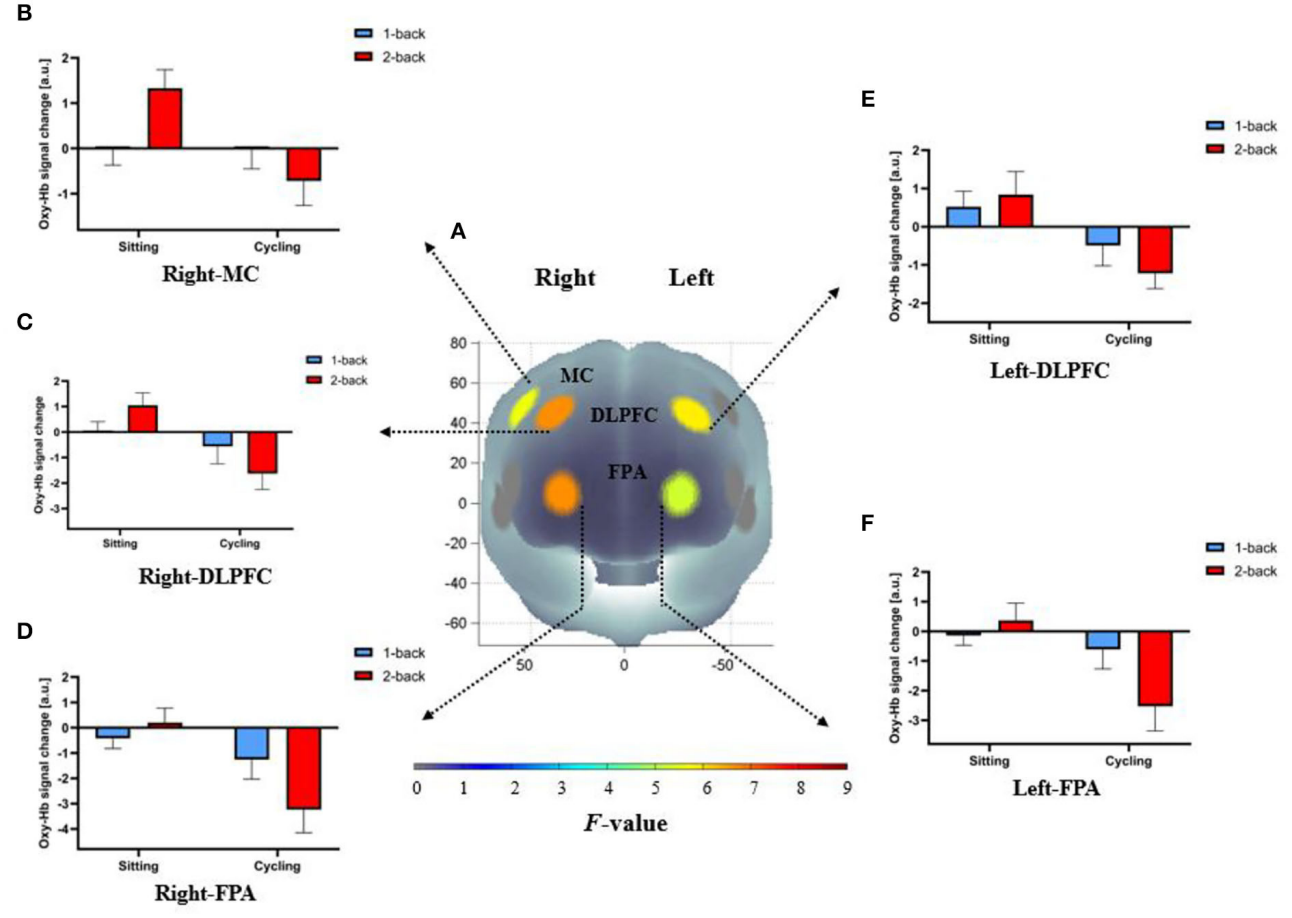
Cortical activation patterns during the n-back task in the sitting and cycling conditions. **(A)** *F*-map of oxy-Hb concentrations reflecting the main effect of the experimental condition. The significant main effect of the experimental condition between sitting and cycling conditions can be seen in the bilateral FPA, DLPFC, and r-MC (*p* < 0.05, FDR-corrected) among the ten regions of interest. *F*-values are displayed according to the color bar. **(B–F)** The mean difference of oxy-Hb concentrations between 1-back and 2-back conditions in both the bilateral FPA, DLPFC, and r-MC for sitting and cycling conditions.

The RM-ANOVA performed on ROI-wise oxy-Hb concentrations revealed that there were significant main effects of the task condition on r-VLPFC [*F*_(1,20)_ = 6.92, $\eta_{\text{p}}^{2}$ = 0.26, *p* < 0.05, FDR-corrected] and l-MTG [*F*_(1,20)_ = 5.04, $\eta_{\text{p}}^{2}$ = 0.2, *p* < 0.05, FDR-corrected]. The results showed that oxy-Hb concentrations were significantly greater in the 2-back task than in the 1-back task in r-VLPFC and l-MTG (Supplementary Table 2). Also, there were significant main effects of the experimental condition on the following: l-FPA [*F*_(1,20)_ = 5.79, $\eta_{\text{p}}^{2}$ = 0.22, *p* < 0.05, FDR-corrected], r-FPA [*F*_(1,20)_ = 9.38, η^2^p = 0.27, *p* < 0.05, FDR-corrected], l-DLPFC [*F*_(1,20)_ = 6.42, $\eta_{\text{p}}^{2}$ = 0.24, *p* < 0.05, FDR-corrected], r-DLPFC [*F*_(1,20)_ = 7.27, $\eta_{\text{p}}^{2}$ = 0.27, *p* < 0.05, FDR-corrected], r-MC [*F*_(1,20)_ = 5.99, $\eta_{\text{p}}^{2}$ = 0.23, *p* < 0.05, FDR-corrected]. The results showed that oxy-Hb concentrations in the cycling condition were significantly lower than those in the sitting condition (l-FPA: 0.15 ± 0.37 vs. −1.57 ± 0.61; r-FPA: −0.11 ± 0.39 vs. −2.26 ± 0.71; l-DLPFC: 0.68 ± 0.45 vs. −0.86 ± 0.35; r-DLPFC: 0.55 ± 0.32 vs. −1.1 ± 0.54; l-MC: 0.54 ± 0.27 vs. −0.74 ± 0.52; r-MC: 0.66 ± 0.29 vs. −0.37 ± 0.43) (Figure 6). The RM-ANOVA performed on ROI-wise oxy-Hb concentrations revealed a significant interaction between the experimental and task conditions in the following: l-FPA [*F*_(1,20)_ = 6.11, $\eta_{\text{p}}^{2}$ = 0.23, *p* < 0.05, FDR-corrected], r-FPA [*F*_(1,20)_ = 9.33, $\eta_{\text{p}}^{2}$ = 0.32, *p* < 0.05, FDR-corrected], r-DLPFC [*F*_(1,20)_ = 5.76, $\eta_{\text{p}}^{2}$ = 0.22, *p* < 0.05, FDR-corrected], and r-MC [*F*_(1,20)_ = 12.11, $\eta_{\text{p}}^{2}$ = 0.38, *p* < 0.05, FDR-corrected]. The results demonstrated that oxy-Hb concentrations in response to the 2-back task in the cycling condition were significantly lower than those in the sitting condition. Oxy-Hb concentrations in response to the 2-back task in r-MC were significantly higher than those in response to the 1-back task in the sitting condition (Supplementary Tables 2, 3).

### The link between exercise-induced arousal, behavioral and fNIRS results

**Figure 7:**
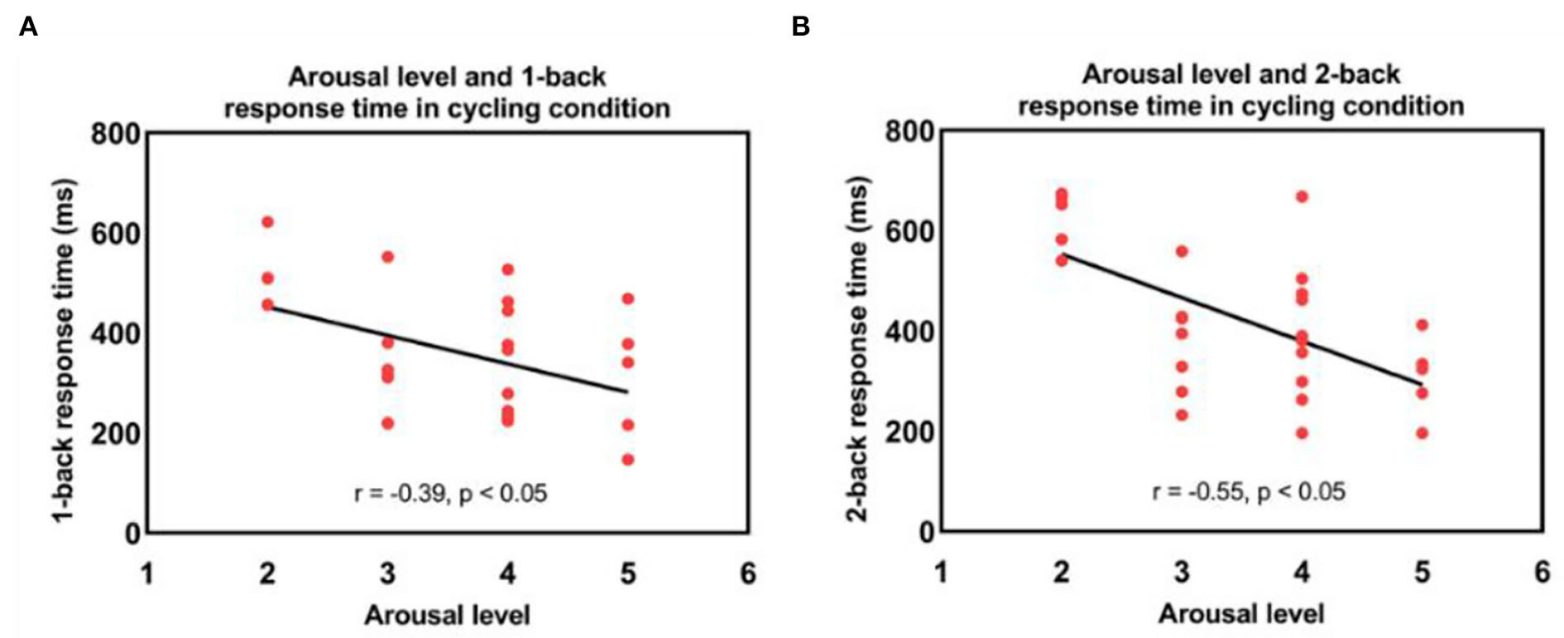
Correlations between the arousal level immediately after exercise and **(A)** 1-back and **(B)** 2-back response times in the cycling condition.

The RM-ANOVA was conducted to examine the changes in arousal level. The results showed that there was a significant interaction between condition and session factors [*F*_(1,27)_ = 9.31, *p* < 0.05] and the main effects for condition [*F*_(1,27)_ = 26, *p* < 0.001] and the sessions [*F*_(1,27)_ = 29.21, *p* < 0.001]. There was no difference in pre-session arousal levels between the sitting and cycling conditions [*F*_(1,27)_ = 3.58, *p* > 0.05]. Furthermore, the arousal level increased significantly immediately after moderate-intensity exercise [*F*_(1,27)_ = 60.09, *p* < 0.001]. Post-session arousal levels for cycling were significantly higher than post-session arousal levels for the sitting condition [*F*_(1,27)_ = 46.27, *p* < 0.001]. In addition, 1-back and 2-back RTs were negatively correlated with exercise-induced arousal levels in the cycling condition (r = −0.39, r = −0.55, *p* < 0.05, respectively, Figure 7). The negative correlation between arousal level and RT indicated that improved working memory performance was associated with elevated level of arousal. There was, however, no significant interaction or main effect for accuracy.

To examine the association between the cycling condition-related response time shortening (1-back and 2-back) and cortical activity in the bilateral FPA, DLPFC, and r-MC, the McNemar test was adopted to assess the correspondence between the two incidences (Stegman, 1989; Yanagisawa et al., 2010). The results demonstrated that the coincidence frequencies for the bilateral FPA, DLPFC, and r-MC were not statistically significant (all *p* > 0.05). Therefore, the present results indicate that the improved working memory performance demonstrated in RT and the bilateral FPA, DLPFC, and r-MC activations were not significantly coincided.

## Discussion

This study examined the concurrent performance of the n-back task and cortical activity during acute moderate-intensity exercise in young adults. It was demonstrated that the RT in the n-back task was facilitated without sacrificing the accuracy in the cycling condition. The fNIRS results indicated that, compared with the sitting condition, the cycling condition resulted in lower oxy-Hb concentrations in the bilateral FPA, DLPFC, and r-MC.

In this study, the concurrent performance of the n-back task was improved during moderate-intensity exercise, as indicated by a faster RT. Currently, only a limited number of studies have investigated the effects of exercise on the concurrent performance of working memory in young adults. Martins et al. (2013) found that the performance of the paced auditory serial addition task (PASAT) and Sternberg task performance were improved during moderate-intensity exercise. Komiyama et al. (2017) also demonstrated a positive effect on working memory measured by spatial delayed response task (Spatial DR) during moderate-intensity exercise. However, some studies observed that working memory was not changed in this exercise intensity (Audiffren et al., 2009; Lambourne et al., 2010; Komiyama et al., 2015, 2020). The discrepancy may be due to the characteristics of varied working memory tasks. Exercise duration may also moderate the exercise-working memory relationship. For instance, some studies with a relatively short duration have shown positive effects on working memory (Martins et al., 2013; Komiyama et al., 2017), whereas no effects were observed in a relatively long duration (30 min or more) (Audiffren et al., 2009; Lambourne et al., 2010; Komiyama et al., 2015).

The arousal measured by FAS significantly increased to a moderate level during cycling. The RT of the n-back task was negatively associated with arousal level, suggesting that working memory performance improved after the rise of arousal during the exercise. Previous studies have suggested an inverted U-shaped relationship between arousal and information processing (Tomporowski, 2003; Lambourne and Tomporowski, 2010). Therefore, the current study supported that exercise-induced elevation of arousal level may, in part, account for the faster RT during the cycling condition. In addition, the catecholamine hypothesis posits that increased phasic release of dopamine and norepinephrine during moderate-intensity exercise enhances cognitive performance, which provide another plausible explanation of the processes involved in facilitating working memory performance during moderate-intensity exercise (McMorris, 2016, 2021).

Concerning the findings of fNIRS, the in-task oxy-Hb concentrations in the bilateral FPA, DLPFC, and r-MC were lower in the cycling condition, which suggested that cortical activation was reduced in those regions during the moderate-intensity exercise. The underlying mechanisms remain unclear. A motor and cognitive dual-task condition is considered more complex and requires both motor and cognitive brain resources (Maidan et al., 2021). It is plausible that simultaneously performing motor and cognitive tasks may lead to competition and reallocation of metabolic and attentional resources. Since the neural circuitry involved in initiation, control, and maintenance of movements requires considerable metabolic resources (Friedman et al., 1982; Lambourne and Tomporowski, 2010), more brain resources were reallocated in the primary motor cortex during the moderate-intensity exercise. However, it remains to be investigated why oxy-Hb concentrations were decreased in the bilateral FPA, DLPFC, and r-MC but not in the other regions.

The current results support those of Lucas et al. (2012) and Schmit et al. (2015). They found that inhibitory control performance was improved during high-intensity exercise despite a decrease in cerebral oxygenation in the PFC. One of our previous studies (Huang et al., 2019) also found that self-paced low-intensity cycling resulted in lower in-task oxy-Hb concentrations in the FPA and DLPFC. Conversely, the present findings are in contrast with a previous study by Stone et al. (2020), which examined cognitive flexibility during graded exercise in military personnel. They found that PFC oxygenation significantly rose across exercise intensities. Similarly, Tempest and Reiss (2019) measured working memory performance using the n-back task under different exercise intensities. They found significant activation in the left PFC while cycling at moderate intensity. Therefore, the cortical hemodynamic changes while simultaneously performing cognitive and motor tasks remain to be elucidated in future studies.

The current study was among the first few ones to examine the in-task cortical activity during aerobic exercise by fNIRS. This study also has some limitations. First, due to the limitation of fNIRS measurement, the set-up of source and detector probes primarily covered the PFC and part of the pre-motor region. The cortical activity in parietal regions and primary motor cortex was not monitored during the task. Therefore, the relationship and interaction of cortical activity between the PFC and other brain regions were not examined. Second, the current study's duration of simultaneously performing cognitive tasks and cycling was about 8 minutes. Potential dynamic changes in working memory and cortical activity during prolonged exercise need investigation in future studies. Third, the study was conducted on young adults; therefore, the findings cannot be directly generalized to other age groups. Future studies are needed to verify the findings in broader age groups.

## Conclusions

The findings suggested that the concurrent performance of working memory was improved during acute aerobic exercise, as indicated by faster RT. However, in-task cortical activity was decreased in bilateral FPA, DLPFC, and r-MC.

## Data availability statement

The original contributions presented in the study are included in the article/Supplementary material, further inquiries can be directed to the corresponding author/s.

## Ethics statement

The studies involving human participants were reviewed and approved by Institutional Review Board for Human Research Protections, Shanghai Jiao Tong University. The patients/participants provided their written informed consent to participate in this study.

## Author contributions

KZ and TH: conceptualization and writing—original draft preparation. KZ, YC, TH, and ZD: methodology. KZ and ZD: formal analysis. KZ, YC, and ZD: resources. KZ, ZD, JQ, YC, SL, and TH: data collection and writing—review and editing. KZ, SL, and TH: data curation. TH: supervision and project administration. All authors have read and approved to the submitted version of the manuscript.

## Conflict of interest

The authors declare that the research was conducted in the absence of any commercial or financial relationships that could be construed as a potential conflict of interest.

## Publisher's note

All claims expressed in this article are solely those of the authors and do not necessarily represent those of their affiliated organizations, or those of the publisher, the editors and the reviewers. Any product that may be evaluated in this article, or claim that may be made by its manufacturer, is not guaranteed or endorsed by the publisher.
