# Supplementary material for: Changes in working memory performance and cortical activity during acute aerobic exercise in young adults
Source: Front Behav Neurosci. 2022 Aug 2;16:884490. doi: 10.3389/fnbeh.2022.884490 (PMC9379142; doi:10.3389/fnbeh.2022.884490)
Supplement: Supplementary file 1 [file Data_Sheet_1.docx]

**Supplementary Table 1** **|** Estimated MNI coordinates for the channel using probabilistic registration.

| **Channel** | **X** | **Y** | **Z** |
| --- | --- | --- | --- |
| 1 | -32.697 | 61.383 | -7.378 |
| 2 | -10.109 | 67.569 | -4.458 |
| 3 | -58.149 | 18.039 | -10.210 |
| 4 | -62.223 | 5.251 | 4.979 |
| 5 | -47.316 | 47.048 | 1.942 |
| 6 | -52.707 | 37.074 | 2.575 |
| 7 | -56.780 | 24.286 | 17.765 |
| 8 | -46.077 | 41.194 | 22.838 |
| 9 | -34.781 | 59.673 | 3.324 |
| 10 | -33.542 | 53.819 | 24.219 |
| 11 | -12.193 | 65.860 | 6.243 |
| 12 | -54.416 | 12.308 | 34.864 |
| 13 | -43.712 | 29.216 | 39.937 |
| 14 | -36.621 | 15.548 | 54.914 |
| 15 | -30.108 | 44.071 | 39.884 |
| 16 | -23.017 | 30.402 | 54.861 |
| 17 | -9.545 | 44.338 | 48.021 |
| 18 | 38.601 | 16.157 | 55.492 |
| 19 | 46.048 | 28.614 | 41.111 |
| 20 | 56.928 | 11.623 | 35.444 |
| 21 | 59.817 | 17.082 | -8.781 |
| 22 | 64.400 | 4.159 | 6.308 |
| 23 | 14.796 | 67.817 | -4.677 |
| 24 | 35.930 | 61.703 | -7.383 |
| 25 | 16.406 | 65.807 | 6.482 |
| 26 | 37.540 | 59.694 | 3.775 |
| 27 | 36.094 | 53.179 | 25.345 |
| 28 | 12.168 | 44.410 | 48.221 |
| 29 | 25.196 | 31.097 | 55.352 |
| 30 | 32.642 | 43.554 | 40.971 |
| 31 | 49.763 | 46.642 | 2.936 |
| 32 | 48.317 | 40.127 | 24.506 |
| 33 | 54.614 | 36.059 | 3.751 |
| 34 | 59.197 | 23.136 | 18.840 |

**Supplementary Table 2** **|** Oxy-Hb signal changes in all ROIs.

| **ROIs** | **Sitting (1-back)** | **Sitting (2-back)** | **Cycling (1-back)** | **Cycling (2-back)** |
| --- | --- | --- | --- | --- |
| l-FPA | -0.14 | 0.37 | -0.61 | -2.53 |
| r-FPA | -0.42 | 0.20 | -1.27 | -3.24 |
| l-DLPFC | 0.53 | 0.84 | -0.49 | -1.22 |
| r-DLPFC | 0.06 | 1.05 | -0.56 | -1.63 |
| l-VLPFC | 0.01 | 0.89 | -1.53 | -0.63 |
| r-VLPFC | -0.69 | 1.97 | -0.17 | 0.49 |
| l-MTG | -0.39 | 1.45 | -1.30 | 0.52 |
| r-MTG | -1.26 | 0.49 | 0.15 | 0.56 |
| l-MC | 0.46 | 0.61 | -0.19 | -1.30 |
| r-MC | -0.01 | 1.33 | -0.01 | -0.72 |

**Supplementary Table 3 |** Two-way repeated-measures analysis of variance (RM-ANOVA) was applied to assess the main effect (task and experimental) and the interaction (task × experimental).

| ROIs | Experiment Condition | | | Task Condition | | | Interaction effect | | |
| --- | --- | --- | --- | --- | --- | --- | --- | --- | --- |
|  | *F*-value | *p* | *η*^2^_p_ | *F*-value | *p* | *η*^2^_p_ | *F*-value | *p* | *η*^2^_p_ |
| l-FPA | 5.787 | 0.026 | 0.224 | 1.539 | 0.229 | 0.071 | 6.109 | 0.023 | 0.234 |
| r-FPA | 7.379 | 0.013 | 0.270 | 1.089 | 0.309 | 0.052 | 9.326 | 0.006 | 0.318 |
| l-DLPFC | 6.423 | 0.020 | 0.243 | 0.328 | 0.573 | 0.016 | 1.467 | 0.240 | 0.068 |
| r-DLPFC | 7.274 | 0.014 | 0.267 | 0.008 | 0.928 | 0.000 | 5.758 | 0.026 | 0.224 |
| l-VLPFC | 3.670 | 0.070 | 0.155 | 1.800 | 0.195 | 0.083 | 0.001 | 0.978 | 0.000 |
| r-VLPFC | 0.579 | 0.456 | 0.028 | 6.921 | 0.016 | 0.257 | 3.414 | 0.079 | 0.146 |
| l-MTG | 1.139 | 0.299 | 0.054 | 5.039 | 0.036 | 0.201 | 0.000 | 0.995 | 0.000 |
| r-MTG | 0.803 | 0.381 | 0.039 | 2.174 | 0.156 | 0.098 | 0.803 | 0.381 | 0.039 |
| l-MC | 5.046 | 0.036 | 0.201 | 1.067 | 0.314 | 0.051 | 1.566 | 0.225 | 0.073 |
| r-MC | 5.993 | 0.024 | 0.231 | 0.606 | 0.445 | 0.029 | 12.114 | 0.002 | 0.377 |

Note that FDR adjusted *p*-value (q-value) < 0.05.
